# Gandou Decoction Decreases Copper Levels and Alleviates Hepatic Injury in Copper-Laden Hepatolenticular Degeneration Model Rats

**DOI:** 10.3389/fphar.2020.582390

**Published:** 2020-10-28

**Authors:** Na Wang, Meimei Cheng, Xueyan Zhang, Hongfei Wu, Huan Wu, Shijian Cao, Peng Wu, An Zhou

**Affiliations:** ^1^ The Experimental Research Center, Anhui University of Chinese Medicine, Hefei, China; ^2^ Department of Pharmacy, West Anhui Health Vocational College, Liuan, China; ^3^ Anhui Province Key Laboratory of Chinese Medicinal Formula, Hefei, China; ^4^ The First Affiliated Hospital of Anhui University of Chinese Medicine, Hefei, China

**Keywords:** hepatolenticular degeneration, Wilson’s disease, Gandou decoction, copper content, oxidative stress

## Abstract

**Objective:** This study was designed to investigate the therapeutic efficacy and underlying mechanisms of Gandou Decoction (GDD) in copper-laden hepatolenticular degeneration (HLD) model rats.

**Methods:** In this study, high-performance liquid chromatography (HPLC) fingerprint analysis and eight representative active components were simultaneously measured for quality control of GDD. The therapeutic effect of GDD in HLD was studied by constructing a rat model of copper-laden HLD. The copper levels in the liver, serum, urine, and feces were quantified by atomic absorption spectrophotometry (AAS). Subsequently, UV-Vis spectrophotometry was used to study the coordination ability of copper ion (Cu^2+^) with six representative active components in GDD to explore its potential copper expulsion mechanism. Serological indexes including alanine aminotransferase (ALT), aspartate aminotransferase (AST), and alkaline phosphatase (AKP) were evaluated. Hepatic indicators including superoxide dismutase (SOD), glutathione (GSH), and the total antioxidant capacity (T-AOC) were determined. Moreover, the liver tissue was stained with hematoxylin-eosin to observe the histological changes.

**Results:** Thirty characteristic fingerprint peaks were used to assess the similarities among 10 samples and showed the similarity was >0.98, indicating a good correlation among the common peaks. Simultaneous quantification of eight markers in GDD was then performed to determine the consistency of quality. GDD could decrease the serum and hepatic copper levels by increasing the urinary and fecal copper content in copper-laden rats. Meanwhile, the results of UV-Vis absorption studies show that six representative active ingredients in GDD can coordinate with Cu^2+^, indicating that complexing copper removal may be a potential mechanism for GDD to play a role in copper removal. Serum hepatic enzyme markers AST, ALT, and AKP activities and antioxidant enzyme SOD, T-AOC activities, and GSH level in hepatic tissue showed the protection of GDD against liver injury induced by excessive copper. Additionally, the hepatoprotective effect of GDD was also evidenced by the results of the liver histological evaluation.

**Conclusions:** This study suggested that GDD could reduce the serum and hepatic copper levels through promoting urinary and fecal copper excretion in copper-laden rats. At the same time, GDD could alleviate hepatic injury by inhibition of oxidative stress.

## Introduction

Hepatolenticular degeneration (HLD), also known as Wilson’s disease (WD), was first described by Kinnear Wilson in 1912 (Wilson, 1912). As an autosomal recessive genetic disorder, HLD was characterized by impaired copper transport and excretion due to the mutation of the ATP7B gene, which encodes a copper-binding ATPase (Ferenci, 2006; Hedera, 2017). The lifelong prevalence of HLD is estimated at 1:30,000, but a recent study on the frequency of abnormal genes indicates that the prevalence is rising (1:7,026) (Coffey et al., 2013). In the process of hepatolenticular degeneration, defects in the excretory pathway of the liver and impediments to the excretion of copper into bile lead to copper accumulate in the liver and other tissues, which in turn causes extensive damage to organs (Brewer and Yuzbasiyan‐Gurkan, 1992). The liver is one of the primary target organs of copper. The increasing copper content in the liver accelerates the rate of physiological free radical reactions and further leading to oxidative stress (Musacco-Sebio et al., 2014). Sustained oxidative stress in liver cells accelerates apoptosis and necrosis by destroying lipids, proteins, and DNA (Yang et al., 2016). These modifications eventually result in liver dysfunction (Cichoż-Lach and Michalak, 2014).

As one of the few genetic neurodegenerative disorders, HLD can be controlled by drug therapy, and the current standard treatment for HLD includes the use of the chelating agents D-penicillamine, trientine, dimercaptosuccinic acid, dimercaptopropanol, sodium dimercaptosulphonate, and calcium disodium edetate. (Walshe, 1956; Li et al., 2016; Liu et al., 2017). Unfortunately, the most frequently used pharmacological agents are associated with multiple adverse effects, such as neurological problems, swelling and inflammation of the kidneys, and hematological abnormalities, which limited their clinical application (Boga et al., 2017). Gandou Decoction (GDD), originated from the experienced prescription of a Traditional Chinese medicine practitioner, has been used in clinics to treat HLD in china for more than 40 years (Hu et al., 2002). GDD was formulated from six herbal medicines, namely *Rheum palmatum* L. (Chinese name: Dahuang), *Coptis chinensis* Franch. (Chinese name: Huanglian), *Curcuma longa* L. (Chinese name: Jianghuang), *Lysimachia christinae* Hance (Chinese name: Jinqiancao), *Alisma orientale* (Sam.) Juzep. (Chinese name: Zexie), *Panax notoginseng* (Burk.) F. H. Chen (Chinese name: Sanqi). Various studies have shown that GDD has the well-documented curative effects of detoxification, heat-clearance, diuretic activity, blood circulation activation, and can be effective in treating HLD, furthermore, its toxic side effects are relatively small (Xue et al., 2007). Clinical studies have demonstrated that the use of GDD in the treatment of HLD can not only significantly increase the amount of copper excreted in 24 h, but also improve the clinical symptoms and signs of patients, which can promote the transformation of the disease to a favorable direction (Xu et al., 2012). Furthermore, GDD has been recommended as the basic prescription for conventional treatment of HLD by “The Diagnosis and Treatment Guidelines of WD in China, in view of its remarkable clinical efficacy”. In our previous studies, 96 compounds including anthraquinones, flavonoids, alkaloids, triterpenoid saponins, protostane triterpenoids, were preliminarily identified in GDD by UPLC-Q-TOF-MS (Xu et al., 2019). On this basis, we further found that anthraquinones, curcuminoids, and protostane triterpenoids were the major components in rat liver. (Xu et al., 2020). In a metabolomics study, we also found that GDD may play a certain therapeutic role by regulating multiple pathways such as lipid metabolism, amino acid metabolism, and sugar metabolism (Cheng et al., 2018). Removing copper and inhibiting oxidative stress are important medical treatments in HLD patients. Although the fact that GDD has a remarkable effect on HLD, the therapeutic effects of GDD on the HLD needed to be further studied. Therefore the present study was designed to assess the therapeutic effects of GDD on copper-laden hepatolenticular degeneration model rats and further explore its underlying mechanism.

## Materials and Methods

### Reagents and Chemicals

*Rheum palmatum* L. (Bach number: 20180106), *Coptis chinensis* Franch. (Bach number: 20180101), *Curcuma longa* L. (Bach number: 20180120), *Lysimachia christinae* Hance (Bach number: 20180113), *Alisma orientale* (Sam.) Juzep. (Bach number: 20180107), *Panax notoginseng* (Burk.) F. H. Chen (Bach number: 20180109) were purchased from Beijing Tongrentang Pharmacy Co., Ltd. (Hefei, China) and verified by Professor Nianjun Yu (a plant taxonomist from Department of Pharmacognosy of Anhui University of Chinese Medicine). All of the samples had been deposited at the Herbarium of Anhui University of Chinese Medicine, Hefei, China (Herbarium code: ACM, voucher numbers: 18056, 18031, 18059, 18070, 18052, 18067). The details of the drug materials are given in Supplementary Table S1. The chemical composition information of GDD and the chemical contribution of individual herbs to the whole decoction were reported previously (Xu et al., 2019). The quality control information of GDD is described in this article. The standard chemicals which include kaempferide, rhein, chrysophanol, aloe-emodin were supplied by Beijing Beina Chuanglian Biotechnology Research Institute (Being, China). Physcion, emodin, quercetin were purchased from National Institutes for Food and Drug Control (Being, China). Curcumin was prepared by the laboratory. The purity of each reference substance was above 98%. Other solvents and reagents were analytical or HPLC grade. Assay kits were used for the determination of alanine aminotransferase (ALT), aspartate aminotransferase (AST), total protein (BCA), glutathione (GSH), superoxide dismutase (SOD), alkaline phosphatase (AKP) and total antioxidant capacity (T-AOC) were purchased from Jiancheng Bioengineering Institute (Nanjing, China).

### Preparation of Gandou Decoction

According to the prescription, a mixture of *Rheum palmatum* L. (100 g), *Coptis chinensis* Franch. (100 g), *Curcuma longa* L. (100 g), *Lysimachia christinae* Hance (120 g), *Alisma orientale* (Sam.) Juzep. (120 g), *and Panax notoginseng* (Burk.) F. H. Chen (15 g) were crushed with a high-speed pulverizer. After evenly mixed, the mixture was diluted 8-fold in water and refluxed two times, each for 1 h. The extracted solution was filtered and combined, then evaporated to a certain volume under reduced pressure and vacuum freeze-dried into a powder with a yield of 15.3%. The freeze-dried powder of GDD was stored in a refrigerator at 4°C for future chromatographic analysis and biological research. Ten batches were prepared and named S1–S10.

### High-Performance Liquid Chromatography and Fingerprint Analysis

Chromatography fingerprints and content determination methods of the GDD were performed with an Agilent-1260 HPLC with an Agilent Eclipse Plus SB-C18 (4.6 mm × 250 mm, 5 µm) analytical column (Agilent Technologies, MA, United States). The column oven temperature was maintained at 30°C. The gradient elution of 0.1% formic acid water (A) and acetonitrile (B) was used with the gradient procedure as follows: 10–25% B in 0–20 min; 25–26% B in 20–28 min; 26–27% B in 28–30 min; 27–30% B in 30–34 min; 30–100% B in 34–52 min; 100–100% B in 52–55 min. The flow rate was 1.0 ml/min, and the injection volume was 3 μL, detection wavelength is 260 nm. Eight reference substances including quercetin, kaempferide, rhein, chrysophanol, physcion, curcumin, emodin, and aloe-emodin, were used for qualitative and quantitative analysis. The Standard stock solutions were prepared by dissolving accurately in methanol and were stored in a 10 ml volumetric flask. Additionally, the freeze-dried powder of GDD was dissolved in methanol to prepare a stock solution with a concentration of 0.035 g/ml and ultrasonic extraction was performed for 20 min.

Before the HPLC analysis, all the test samples were filtered through a 0.22 µm membrane filter. The HPLC fingerprint standard of GDD was recorded and compared to the eight reference substances. The content of the eight constituents of GDD from ten different batches was quantified according to the area under the curve of the corresponding peak.

### Animals and Experimental Design

Adult male Sprague-Dawley (SD) rats, weighing around 180–220 g were obtained from the Anhui Medical University (Hefei, China, No: SCXK (wan) 2017-001). All animals were raised in a controlled environment (room temperature: 24 ± 2°C, relative humidity: 60 ± 10% and 12 h light-dark cycle). After 7 days of normal feeding, forty-eight rats were randomly divided into six groups (n = 8 per group): normal control group; model group; GDD-low dose group (0.65 g/kg/day); GDD-medium dose group (1.30 g/kg/day); GDD-high dose group (2.6 g/kg/day); and D-penicillamine (PA) as the positive control group (0.048 g/kg/day). Rats in the copper-laden model group were administered a CuSO_4_ solution orally at a dose of 200 mg/kg/day by gavage over the period of 60 days, while the control group was given the same dose of distilled water (200 ml/kg/day) by gavage (Kumar et al., 2016). The GDD and PA groups were treated in the same manner as the copper-laden rats to establish the model. However, these groups of rats were treated with PA and the different doses of freeze-dried powder of GDD in the last 30 days. All the freeze-dried powder of GDD was dissolved in 0.5% CMC-Na solution when administered. The control and model rats were given an equivalent volume of 0.5% CMC-Na solution in the same way. After two months, copper concentrations in urine and liver were measured to prove that the model group was a mature copper-laden model. All animal experiments were performed in accordance with the laboratory animal care and use specifications approved by the Animal Ethics Committee of Anhui University of Traditional Chinese Medicine.

### Sample Collection

After 60 days of feeding, the rats were placed into a metabolic cage to gather the urine and feces for 24 h. Subsequently, each rat was anesthetized with 3% pelltobarbitalum natricum. The liver tissues were removed and then stored at −80°C. Blood was collected by the abdominal aortic method and stored at room temperature for 1.5 h. Blood and urine samples were processed by centrifugation at 4,000 rpm for 10 min and then stored at −80°C until the next analysis.

### Copper Content Examination

The total volume of urine specimens was recorded and then centrifuged. From each urine sample, 2 ml was stored in a 5 ml polyethylene tube. Then, the concentrated HNO_3_ was added to the tube at the ratio of 100:1. The blood sample had the same processing method as the urine sample. The content of urinary copper was determined and calculated after shaking. The fecal samples were dried in a vacuum freeze-dryer for 24 h, and then the freeze-dried feces samples were ground into powder and mixed, and stored in a desiccator. 500 mg of liver tissue and lyophilized feces were removed into the conical flasks and then treated with microwave digestion. The content of copper in the liver, serum, urine, and feces was determined by AAS (Thermo Corp., United States).

### Study on the Coordination Ability of Six Representative Components in Gandou Decoction With Cu^2+^

To explore whether the six representative components of GDD coordinate with Cu^2+^, we measured UV-Vis spectroscopy of components with various amounts of the copper complex. The absorbance measurements were performed by keeping the compound concentration constant, and then gradually increase the concentration of Cu^2+^. All samples were adjusted to pH 7.4 with 0.1 M Na_2_HPO_4_ and NaH_2_PO_4_ and incubated at 37°C for 15 min. The spectra were recorded in the range of 200–400 nm by Shimadzu UV-2550 spectrophotometer (Suzhou, China).

### Determination of Coordination Ratios of Copper Complexes

The mole ratio method (Fu and Chen, 2019) was applied to determine the formula for the complexes between six compounds of GDD and Cu^2+^. By keeping the concentration of the monomer compound constant, we added different concentrations of copper ions at different molar ratios (1:10, 1:4, 1:2, 1:1, 1.5:1, 2:1, 2.5:1, 3:1). After the reaction is balanced, the absorbance is measured by UV spectrophotometry at the maximum wavelength. Then make a molar ratio-absorbance curve, and the molar ratio corresponding to the turning point of the obtained curve is the coordination ratio of the copper complex.

### Determination of Equilibrium Constants

Equilibrium shift method was used to determine the equilibrium constant (Zhou et al., 2004). 5 ml of 10 μmol/L Cu^2+^ solution was accurately pipetted into a 10 ml volumetric flask, then different volumes of high concentration reserve solution were added respectively and finally use methanol to volume to the scale. Shake well and measure the absorbance value after the reaction is balanced and stable. According to Lamber-Beer law, under the condition that the concentration of the compound is much greater than the concentration of Cu^2+^, this formula can be derived to calculate the equilibrium constant of the complex, and the calculation formula is as follows: ln[(*A*_0_ − *A*_*e*_)/(*A*_*e*_ − *A*_*∞*_)] = *n*∙ln*C* + ln*K*.

According to this formula, the linear coordination of ln[(*A*_0_ − *A*_*e*_)/(*A*_*e*_ − *A*_*∞*_)] to ln*C* can calculate the coordination number *n* and equilibrium constant *K* of the complex. That *A*_0_ represents the absorbance value of the system when the concentration of Cu^2+^ is 0; *A*_*e*_ represents the absorbance value of the system when the concentration of the reference substance is *C*, *A*_*∞*_ represents the system when the concentration of the reference substance is an infinite multiple of the Cu^2+^ concentration.

### Determination of Thermodynamic Constants

Generally, the forces that facilitate the interaction of macromolecules with small ligands include hydrogen bonding, electrostatic interactions, van der Waals forces, and hydrophobic forces. This acting force may be predicted by knowing the value of the enthalpy change ($\Delta_{r}H_{m}^{\theta }$) and entropy change ($\Delta_{r}S_{m}^{\theta }$). According to the theory of chemical equilibrium thermodynamics, under different temperature conditions, the molar enthalpy change and molar entropy change of the system can be obtained by the Van’t Hoff equation. The calculation formula is as follows:

$$\ln K=-\Delta_{r}H_{m}^{\theta }/\mathrm{RT}+\Delta_{r}S_{m}^{\theta }/R.$$

In addition, according to the Gibbs free energy formula, the Gibbs free energy variation is calculated, and the calculation formula is:

$$\Delta_{r}G_{m}^{\theta }=\Delta_{r}H_{m}^{\theta }-T\cdot \Delta_{r}S_{m}^{\theta }.$$

### Measurements of Serum Alanine Aminotransferase, Aspartate Aminotransferase, and Alkaline Phosphatase

The serum was collected from blood samples after placing at room temperature for 1.5 h and centrifugation at 4,000 rpm for 10 min (Ki et al., 2013). Serum ALT, AST, and AKP activities were measured with kits according to the manufacturer’s instructions.

### Measurement of Glutathione, Superoxide Dismutase, and Total Antioxidant Capacity in Liver Homogenate

Liver tissues were rapidly homogenized in cold physiological saline at a ratio of 1 g liver/9 ml saline. After centrifugation at 3,000 rpm for 10 min, the antioxidant status of supernatants was immediately determined. (Deng et al., 2012). The activities of antioxidant defense enzymes, SOD and GSH, as well as the level of T-AOC were determined following the instructions provided with the kit. BCA protein detection kit was used to determine protein content. All values were normalized according to the protein content of the same sample.

### Histopathological Studies

The residual part of liver tissues (n = 6 per group) was acquired from the same Lobe were fixed in 4% polycondensation formaldehyde solution for over 24 h, and then dehydrated for 12 h. Subsequently, a microtome was used to prepare 5 mm-thick sections, and then stained with hematoxylin and eosin (H&E). Images of the hepatic sections were captured by an electric microscope.

### Statistical Analysis

Data were expressed as mean ± standard error of the means (S.E.M.) per group. Statistical differences between groups were compared by a one-way analysis of variance (ANOVA), followed by Bonferroni’s multiple comparison test. A difference of *p* < 0.05 was considered statistically significant. The statistical analysis was done by using SPSS-23 (SPSS Inc., Chicago, Illinois, United States) or Graphpad Prism 5.0 software (GraphPad Software, CA, United States).

## Results

### High-Performance Liquid Chromatography Fingerprint of Gandou Decoction

**FIGURE 1:**
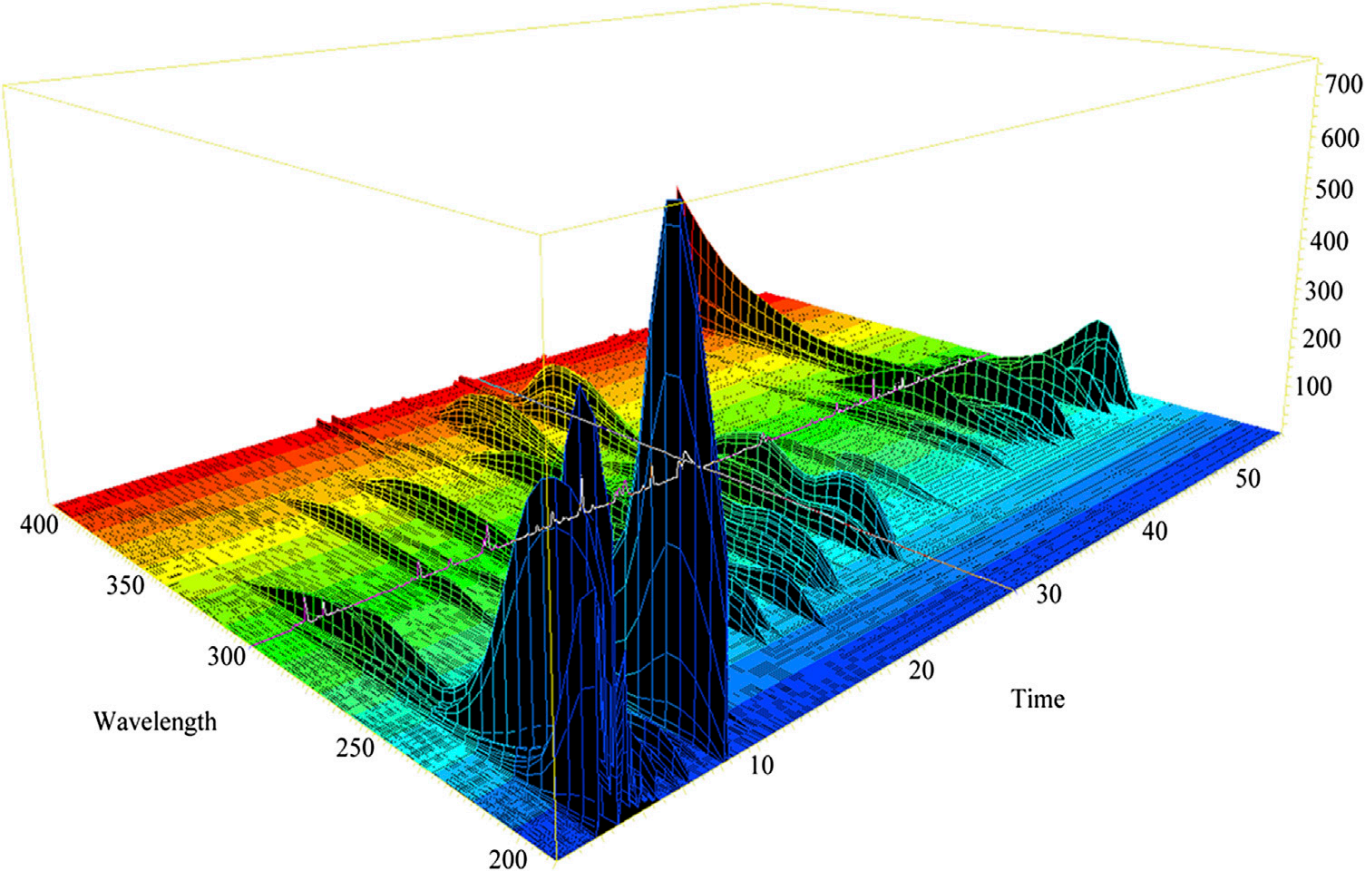
The 3D-HPLC chromatogram of GDD.

**FIGURE 2:**
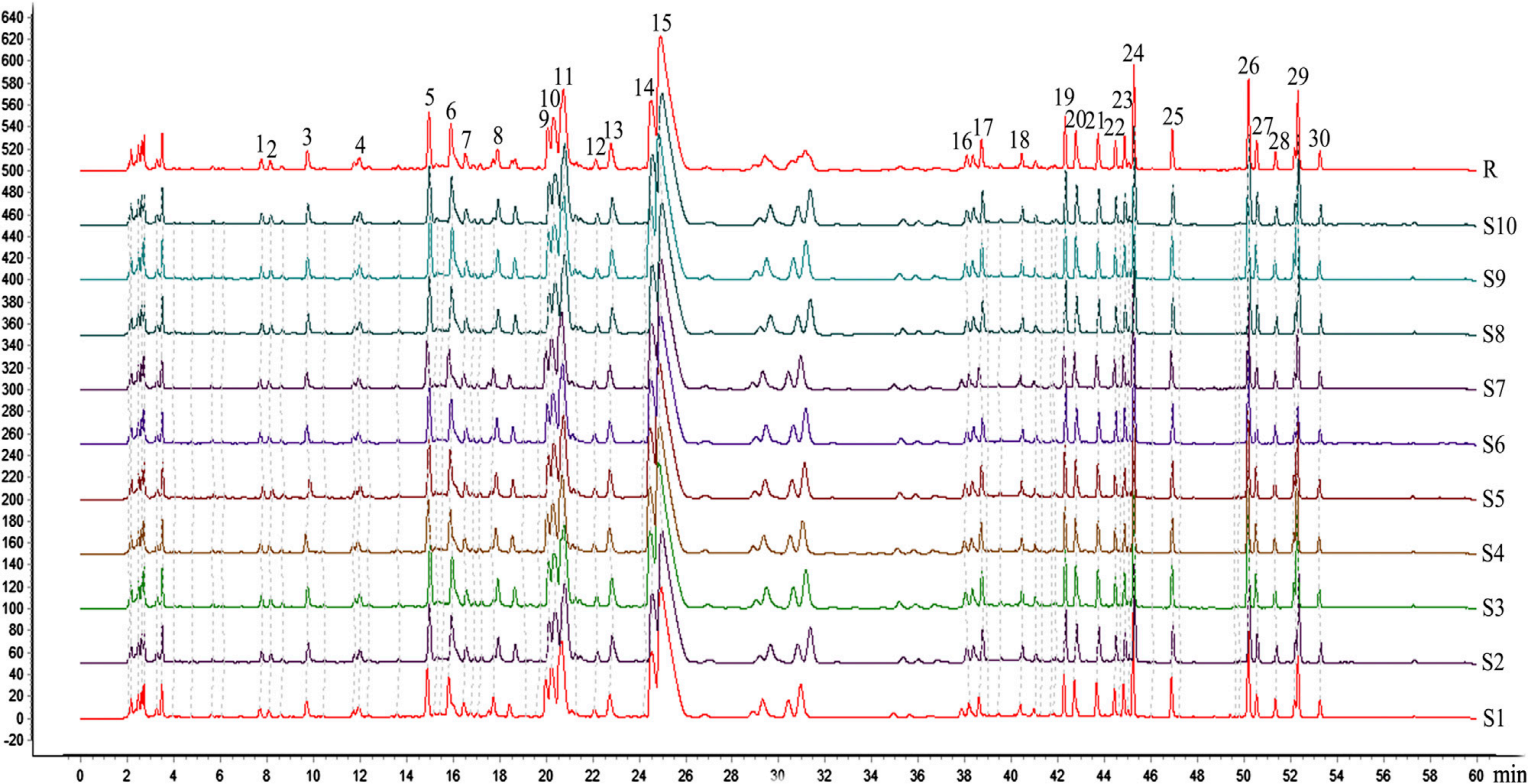
The common pattern diagram of HPLC fingerprint chromatograms of extracts from Gandou Decoction (GDD).

**FIGURE 3:**
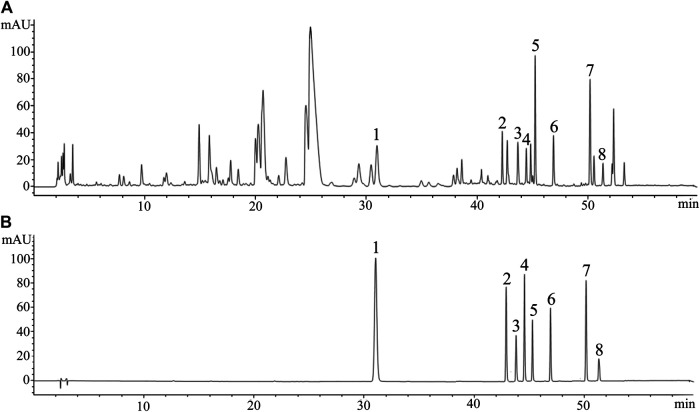
The fingerprint of GDD and mixture of 8 standard products. **(A)** the fingerprint of GDD. **(B)** mixture of 8 standard products. 1) quercetin; 2) aloe-emodin; 3) rhein; 4) kaempferide; 5) curcumin; 6) emodin; 7) chrysophanol; 8) physcion.

3D-HPLC chromatogram of GDD was assayed according to the method described previously. The 3D-HPLC of GDD was shown in Figure 1. To investigate the stability and ensure the quality of the formula, we analyzed the fingerprints of GDD from ten samples (Figure 2). Thirty major peaks were identified in the HPLC fingerprints. All the chromatograms of 10 batches of GDD were matched by the National Pharmacopoeia Committee Chinese Medicine Fingerprint Similarity Evaluation System (Version 2012) Software. As shown in Table 1. The similarity between the fingerprints of the ten samples of GDD and the standard chromatogram was over 0.980, meeting the requirements of the fingerprint. These data suggested that the GDD was of high quality and stable. By comparing with the retention time and UV spectrum of the standard compounds, the quercetin (peak 1), aloe-emodin (peak 2), rhein (peak 3), kaempferide (peak 4), curcumin (peak 5), emodin (peak 6), chrysophanol (peak 7), and physcion (peak 8) were identified (Figure 3). Due to the retention time and height of the shape of peak 13 in the fingerprint are both moderate, this peak can be designated as the reference peak. Therefore, it is convenient to calculate the relative retention time (RRT) and relative peak area (RPA) of each characteristic peak relative to the peak 13. The data of RRT and RPA for these 30 peaks were calculated from this reference peak (peak 13) and the results are shown in Table 2, which can be used to monitor the internal quality of GDD.

**TABLE 1:** The results of similarities of the chromatograms from different batches of GDD.

N0.	Similarity										
	S1	S2	S3	S4	S5	S6	S7	S8	S9	S10	R
S1	1	0.984	0.998	0.988	0.997	0.995	0.985	0.989	0.997	0.994	0.996
S2	0.984	1	0.984	0.984	0.984	0.987	1	0.986	0.986	0.985	0.993
S3	0.998	0.984	1	0.980	0.999	0.997	0.989	0.988	0.997	0.994	0.997
S4	0.988	0.984	0.980	1	0.980	0.980	0.985	1	0.982	0.980	0.990
S5	0.997	0.984	0.999	0.980	1	0.996	0.984	0.980	1	0.993	0.997
S6	0.995	0.987	0.997	0.980	0.996	1	0.991	0.982	0.996	1	0.997
S7	0.985	1	0.989	0.985	0.984	0.991	1	0.984	0.988	0.989	0.993
S8	0.989	0.986	0.988	1	0.980	0.982	0.984	1	0.986	0.985	0.990
S9	0.997	0.986	0.997	0.982	1	0.996	0.988	0.986	1	0.995	0.997
S10	0.994	0.985	0.994	0.980	0.993	1	0.989	0.985	0.995	1	0.997
R	0.996	0.993	0.997	0.990	0.997	0.997	0.993	0.990	0.997	0.997	1

**TABLE 2:** The retention time (t_R_), relative retention time (RRT), peak area (PA) and relative peak area (RPA) of 30 characteristic fingerprint peaks in GDD.

Peak no.	t_R_ (min)	RRT		PA (mVS)	RPA	
		Average	RSD (%)		Average	RSD (%)
1	7.756	0.341	0.56	72.904	0.267	10.39
2	8.143	0.358	0.57	59.030	0.216	9.47
3	9.734	0.427	0.54	145.366	0.532	10.08
4	11.964	0.525	0.30	117.960	0.432	8.56
5	14.959	0.657	0.29	404.900	1.483	8.87
6	15.900	0.698	0.34	450.606	1.650	8.91
7	16.521	0.726	0.25	128.207	0.470	7.53
8	17.707	0.778	0.26	72.2470	0.265	62.67
9	20.061	0.881	0.28	342.690	1.255	7.49
10	20.312	0.892	0.27	636.449	2.331	7.17
11	20.732	0.910	0.26	1206.278	4.418	8.10
12	22.122	0.972	0.27	73.3090	0.268	10.63
13 (S)	22.770	1.000	0.23	273.065	1.000	12.92
14	24.500	1.076	0.17	1023.162	3.747	4.15
15	24.910	1.094	0.21	4522.499	16.562	7.15
16	38.294	1.682	0.19	138.848	0.508	7.55
17	38.701	1.700	0.16	183.776	0.673	16.17
18	40.440	1.776	0.09	87.290	0.320	18.8
19	42.297	1.858	0.07	234.678	0.859	10.66
20	42.761	1.878	0.08	240.609	0.881	7.20
21	43.714	1.920	0.08	212.670	0.779	7.89
22	44.460	1.953	0.06	132.703	0.486	8.46
23	44.853	1.970	0.06	142.171	0.521	8.91
24	45.252	1.987	0.04	483.057	1.769	8.01
25	46.901	2.060	0.05	226.967	0.831	7.29
26	50.171	2.203	0.06	505.276	1.850	7.72
27	50.522	2.219	0.05	156.973	0.575	19.51
28	51.336	2.255	0.07	105.058	0.385	7.11
29	52.295	2.297	0.06	413.034	1.513	23.28
30	53.242	2.338	0.04	102.423	0.375	10.00

### Quantitative Analysis of Samples

As a traditional Chinese medicine compound, multi-index components determination is necessary, which was used to chemically characterize the difference and similarity between different batches, so as to serve for its quality control and comprehensive evaluation. Therefore, the main components of GDD were analyzed quantitatively. The established HPLC quantitative analysis method was verified by linearity, the limit of detection (LOD), the limit of quantitation (LOQ), precision, stability, repeatability, and recovery. As shown in Table 3. The concentration of the analytes demonstrated satisfactory linearity (r^2^ > 0.999) with the peak area within the test ranges. The LOD and LOQ values of eight analytes ranged from 0.21 to 0.48 μg/ml and 0.70 to 1.67 μg/ml. The RSD values of precision, stability, and repeatability of the eight analytes were all less than 3%. The overall recoveries laid between 96.91 and 100.13% with RSD less than 2.22%. All these results indicated that the established HPLC method was linear, sensitive, precise, accurate, and stable enough for the simultaneous quantification of eight major constituents in GDD.

**TABLE 3:** Calibration curves with r value, linear range, the limit of detection (LOD), limit of quantity value (LOQ), precision, reproducibility, stability, and recovery of the eight analytes.

	Calibration curves	r	Linear range	LOD	LOQ	Precision	Repeatability	Stability	Recoveries	
			(µg/ml)	(µg/ml)	(µg/ml)	RSD	RSD		(%, n = 6)	
						(%, n = 6)	(%, n = 6)	(%)	Mean	RSD
Quercetin	y = 4710.0x + 4.9947	0.9994	5.16–51.56	0.48	1.67	1.82	1.89	1.98	99.67	1.58
Aloe-emodin	y = 3634.6x − 0.4983	0.9997	2.60–26.00	0.27	0.83	1.59	2.16	2.86	97.43	0.92
Rhein	y = 1486.2x − 0.4860	0.9995	3.28–32.78	0.33	1.10	2.24	2.36	2.27	98.36	0.94
Kaempferide	y = 3359.4x − 2.9033	0.9996	2.90–29.00	0.31	0.98	2.57	2.97	2.18	98.73	2.09
Curcumin	y = 1334.3x − 4.7807	0.9999	4.09–40.89	0.41	1.36	0.89	2.09	1.02	97.38	2.22
Emodin	y = 2820.5x − 2.0968	0.9998	2.46–24.56	0.21	0.70	1.86	2.75	1.95	96.91	2.02
Chrysophanol	y = 2949.1x − 2.5418	0.9995	3.42–34.22	0.36	1.20	1.18	1.54	1.38	100.13	1.31
Physcion	y = 1031.5x − 1.7041	0.9992	2.89–28.89	0.23	0.79	1.52	2.36	2.06	97.25	1.93

The validated HPLC method was utilized to simultaneously quantify eight constituents of GDD from ten different batches. After calculation, the content of quercetin, aloe-emodin, rhein, kaempferide, curcumin, emodin, chrysophanol, and physcion was shown in Table 4. There was little difference in the content of the components between ten batches. These results indicate that the quality of GDD is stable and uniform.

**TABLE 4:** Content (mg/g) of the eight compounds in ten batches freeze-dried powder samples of GDD.

Batch namber	Content (mean ± SD, n = 3)							
	Quercetin	Aloe-emodin	Rhein	Kaempferide	Curcumin	Emodin	Chrysophanol	Physcion
S1	2.86 ± 0.059	2.76 ± 0.025	5.51 ± 0.033	1.43 ± 0.029	16.72 ± 0.062	3.21 ± 0.066	6.97 ± 0.046	4.45 ± 0.073
S2	2.98 ± 0.01	3.09 ± 0.031	6.89 ± 0.053	2.51 ± 0.045	16.57 ± 0.093	3.96 ± 0054.	7.54 ± 0.029	5.38 ± 0.046
S3	3.32 ± 0.036	2.52 ± 0.062	5.85 ± 0.028	1.24 ± 0.011	17.59 ± 0.071	3.66 ± 0.048	6.68 ± 0.064	4.24 ± 0.039
S4	3.51 ± 0.074	3.21 ± 0.054	7.16 ± 0.044	1.98 ± 0.018	16.58 ± 0.120	3.52 ± 0.015	6.72 ± 0.033	4.36 ± 0.042
S5	2.46 ± 0.016	3.46 ± 0.019	5.37 ± 0.068	1.13 ± 0.022	15.15 ± 0.057	4.04 ± 0.026	6.44 ± 0.019	5.89 ± 0.055
S6	2.97 ± 0.073	2.97 ± 0.083	6.31 ± 0.037	1.82 ± 0.074	15.99 ± 0.063	3.58 ± 0.020	6.17 ± 0.027	4.27 ± 0.028
S7	2.79 ± 0.097	3.19 ± 0.023	7.86 ± 0.024	2.46 ± 0.025	15.27 ± 0.048	4.86 ± 0.043	6.64 ± 0.038	4.28 ± 0.018
S8	3.93 ± 0.0.62	2.31 ± 0.0.35	6.16 ± 0.043	1.88 ± 0.077	14.35 ± 0.076	3.52 ± 0.016	6.38 ± 0.044	5.37 ± 0.046
S9	4.01 ± 0.070	4.49 ± 0.038	5.35 ± 0.064	1.45 ± 0.033	16.25 ± 0.082	4.24 ± 0.024	7.34 ± 0.028	5.99 ± 0.039
S10	2.99 ± 0.057	2.97 ± 0.042	4.93 ± 0.051	2.21 ± 0.014	17.69 ± 0.089	3.55 ± 0.028	7.12 ± 0.053	5.23 ± 0.072

### Effects of Gandou Decoction on Serum and Hepatic Copper Levels of Copper-Laden Model Rats

To further investigate the extent to which GDD may reduce copper levels and improve the symptoms associated with HLD, we used the rat model of copper-laden hepatolenticular degeneration. This is a reliable and convenient rat model that recapitulates HLD-associated phenotypes, including copper accumulation *in vivo* and hepatic injury, which has been used widely as a model to investigate HLD (Kumar et al., 2016).

**FIGURE 4:**
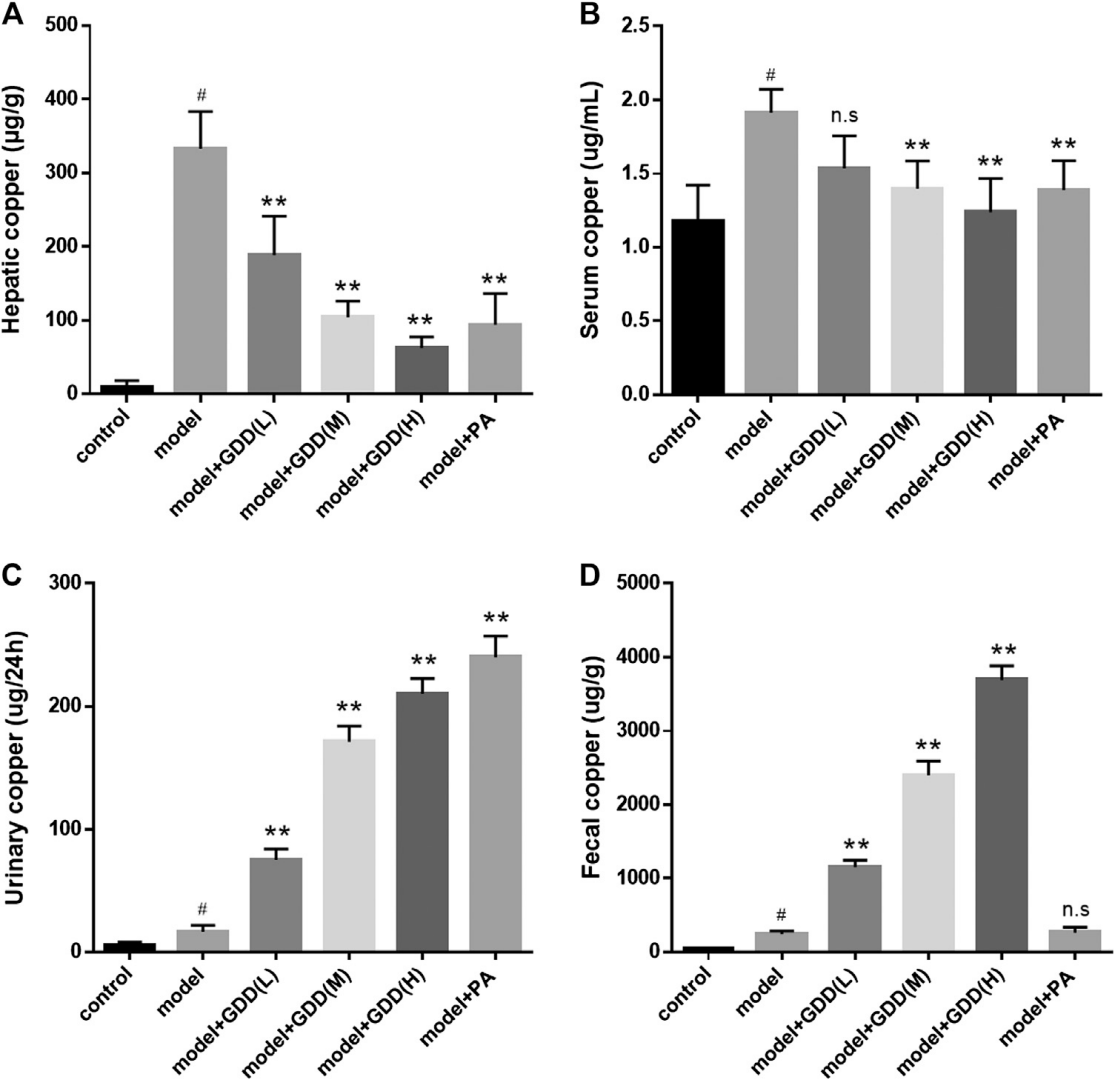
**(A)** Copper content in liver. **(B)** Copper content in serum. **(C)** Copper content in urine. **(D)** Copper content in feces. ^#^ *p* < 0.01, compared with the control group; **p < 0.01, compared with model group; n.s, not significant.

To obtain a quantitative method for the determination of copper levels, the atomic absorption spectrophotometry was used. We determined the copper content in the liver and serum of the rat groups: normal control group; model group; GDD-low dose group (0.65 g/kg/day); GDD-medium dose (1.30 g/kg/day); GDD-high dose group (2.6 g/kg/day); and positive control group. As shown in Figure 4, the atomic absorption spectrum analysis revealed that copper levels were statically higher in the liver (Figure 4A, *p* < 0.01) and serum (Figure 4B, *p* < 0.01) of the copper-laden model rats than those of the control animals. However, when GDD was orally administered to the copper-laden rats, the GDD treated groups had dramatically reduced copper levels in the liver (*p* < 0.01). Meanwhile, the GDD-medium dose (1.30 g/kg/day) and GDD-high dose (2.6 g/kg/day) groups had remarkably reduced copper contents in the serum (*p* < 0.01). In addition, we found that the GDD-high dose (2.6 g/kg/day) was more effective than PA at removing copper from the liver and serum (Figure 4, *p* < 0.01).

### Effects of Gandou Decoction on Urinary and Fecal Copper Levels of Copper-Laden Model Rats

It has been reported that using PA as a “decoppering” agent can promote the excretion of copper in the urine (Humann-Zie-hank and Bickhardt, 2001; Walshe, 2011). Moreover, a new study showed that the DPM-1001 which is a new copper ion chelator can increase the excretion of copper in the feces (Krishnan et al., 2018). Therefore, to investigate the mechanism by which GDD cleared tissue copper levels, we next examined the effects of GDD on the urinary copper content and fecal copper content. Compared to the model rats, the levels of copper in the urine of PA-treated rats were significantly elevated. Similar to the PA treatment, treatment with GDD also resulted in a significant increase in urinary copper levels (Figure 4C, *p* < 0.01). In contrast to model rats, fecal copper levels of GDD-treated copper-laden rats were markedly elevated. Unlike GDD, the treatment of model rats with PA cannot significantly improve fecal copper levels (Figure 4D, *p* < 0.01).

### Study on the Coordination Ability of Six Representative Components in Gandou Decoction With Cu^2+^

**FIGURE 5:**
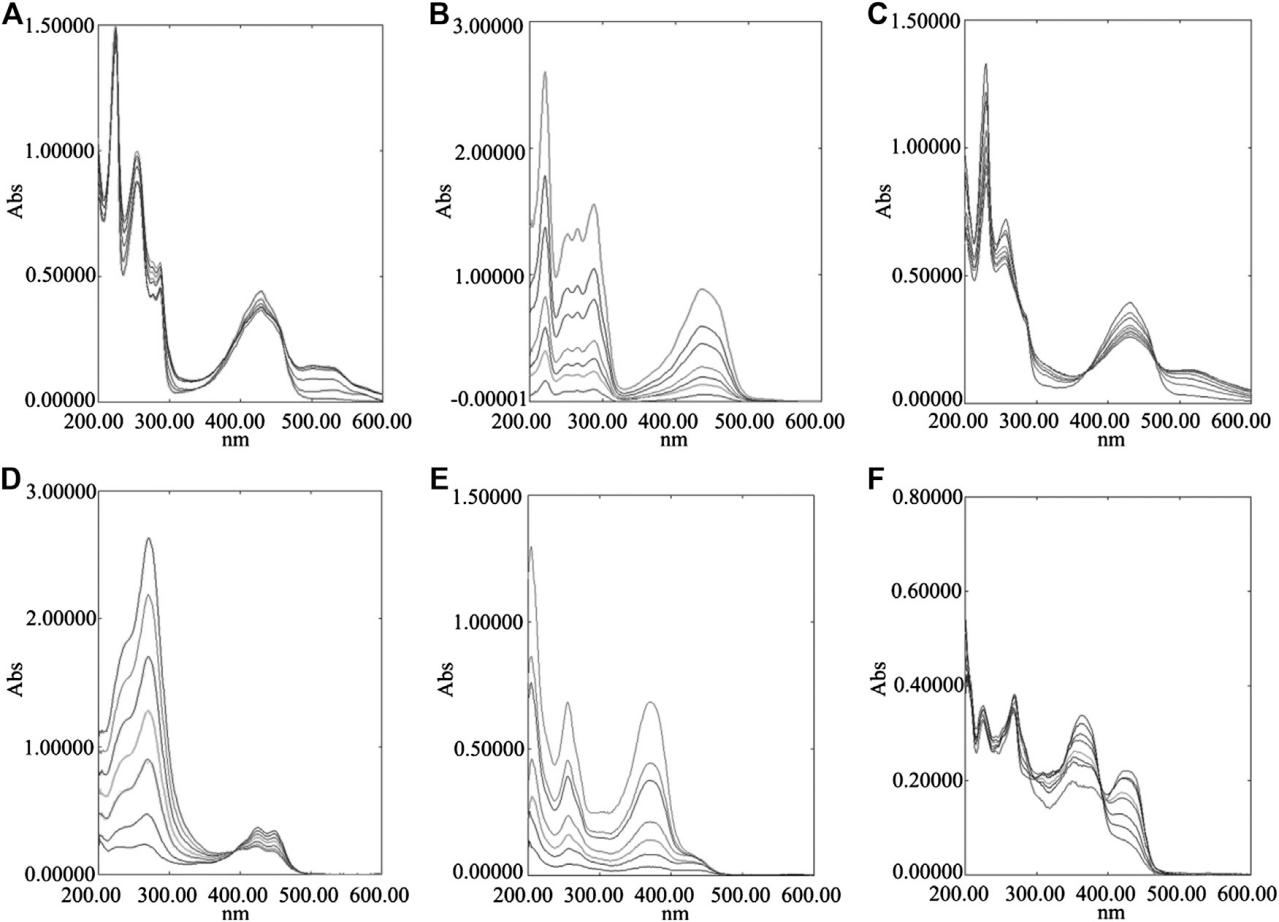
UV-Visible absorption spectra of six compounds coordinated with different concentrations of Cu^2+^. **(A)** chrysophanol; **(B)** emodin; **(C)** rhein; **(D)** curcumin; **(E)** quercetin; **(F)** kaempferide.

Removing copper is the primary treatment for HLD at present. In clinic, metal chelating agents are mainly used for treatment. This kind of medicine can coordinate with Cu^2+^ in the body to form a complex, and then excreted through the urine to achieve the purpose of treatment. Meanwhile, our previous studies on the metabolism spectrum of GDD have shown that the prototype components and metabolites of six representative components (quercetin, kaempferide, curcumin, rhein, chrysophanol, emodin) of GDD can be found in the blood, liver or urine (Xu et al., 2020). These compounds contain phenolic hydroxyl, carboxyl, amino, and other coordination groups, which may provide one or more pairs of lone electrons to coordinate with copper ions containing empty orbitals. Therefore, these six compounds were selected to verify the coordination between the main active components of GDD and Cu^2+^. From Figure 5, it can be observed that with increasing amounts of Cu^2+^ added to the component solution, the intensity of the absorption peak of each compound decreases significantly and redshifts slightly. These results indicate that each component has a stronger coordination effect with Cu^2+^.

### Determination of Coordination Ratios

**FIGURE 6:**
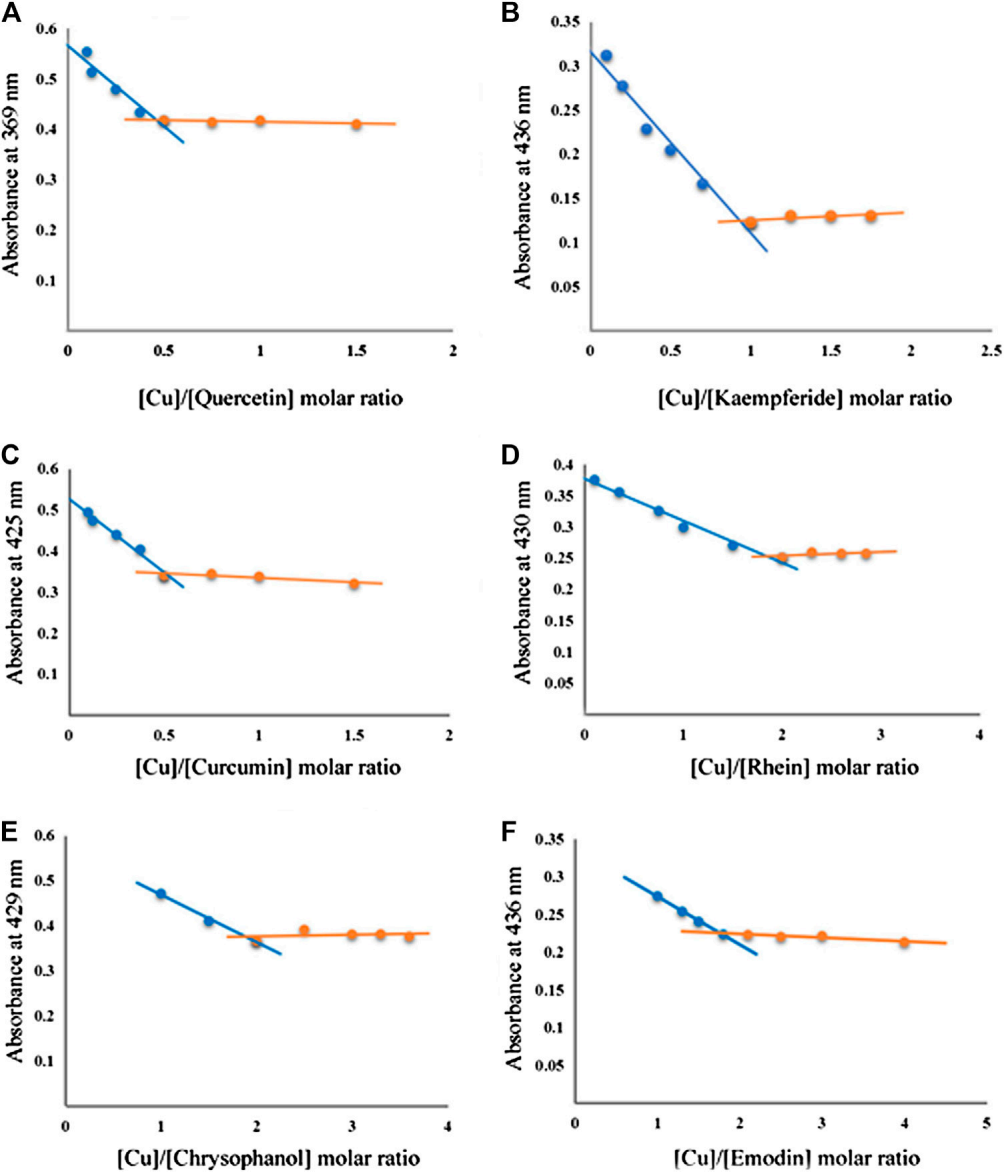
The plot of the absorbance at λ_max_ vs. mole fraction of compounds. **(A)** quercetin; **(B)** kaempferide; **(C)** curcumin; **(D)** rhein; **(E)** chrysophanol; **(F)** emodin.

The coordination ratios of various compounds to Cu^2+^ were determined by UV-Vis spectroscopy. The curve of the maximum absorbance equivalent of Cu^2+^ addition (component: Cu molar ratio) under the tested conditions is shown in Figure 6.

The absorbance plots at 369 nm against the molar fraction of quercetin have a minimum at X quercetin/Cu = 0.5 (Figure 6A), which suggests that the stoichiometric ratio for the complexation of quercetin (flavonoids) and Cu^2+^ is 2:1. According to the same method, the coordination ratio of kaempferide (flavonoids) to Cu^2+^ was calculated as 1:1. The coordination ratio of curcumin to Cu^2+^ was calculated as 2:1. The coordination ratios of anthraquinone compounds (rhein, chrysophanol, emodin) to Cu^2+^ is 1:2.

### Determination of Equilibrium Constants and Thermodynamic Constants

In order to further demonstrate the reaction mechanism of each monomer compound with Cu^2+^, we then conducted a chemical reaction thermodynamic study. The values of *K*, $\Delta_{r}H_{m}^{\theta }$, $\Delta_{r}S_{m}^{\theta }$ and $\Delta_{r}G_{m}^{\theta }$ are summarized in Table 5. The negative value $\Delta_{r}H_{m}^{\theta }$ of indicates that these reactions are exothermic. And the negative value of $\Delta_{r}S_{m}^{\theta }$ indicates that the reaction is a process of entropy decrease, disorder decrease, and order increase. Negative value of $\Delta_{r}G_{m}^{\theta }$ directly provides an inference that the interaction behavior between compounds and Cu^2+^ is spontaneous and thermodynamically favorable.

**TABLE 5:** Coordination equilibrium constants and thermodynamic constants of copper complexes.

	molar ratio (compound/Cu)	K × 10^7^	$\Delta_{r}H_{m}^{\theta }\left(kJ/mol\right)$	$\Delta_{r}S_{m}^{\theta }\left(J\cdot mol^{-1}\cdot K^{-1}\right)$	$\Delta_{r}G_{m}^{\theta }\left(KJ/mol\right)$
Quercetin	2:1	1.616	−11.683	−16.072	−6.894
Kaempferide	1:1	1.459	−17.132	−35.21	−6.641
Curcumin	2:1	7.094	−11.324	−2.567	−10.559
Rhein	1:2	9.063	−14.453	−11.03	−11.166
Chrysophanol	1:2	7.263	−13.125	−8.41	−10.617
Emodin	1:2	1.451	−10.882	−14.28	−6.626

These results showed that all the six compounds in the GDD could coordinate with Cu^2+^ to form stable complexes. Further analysis based on equilibrium constants and thermodynamic constants show that the coordination equilibrium constant K > 10^6^, indicating that the coordination reaction of the copper complex is relatively complete, which is a spontaneous exothermic and entropy reduction process. Comprehensive UV absorption data analysis shows that all compounds had strong coordination ability with Cu^2+^, among which emodin, curcumin, and emodin were the strongest. Therefore, these compounds are the main active ingredients that may play a role in copper removal in GDD.

### Determination of Serological Indexes

**FIGURE 7:**
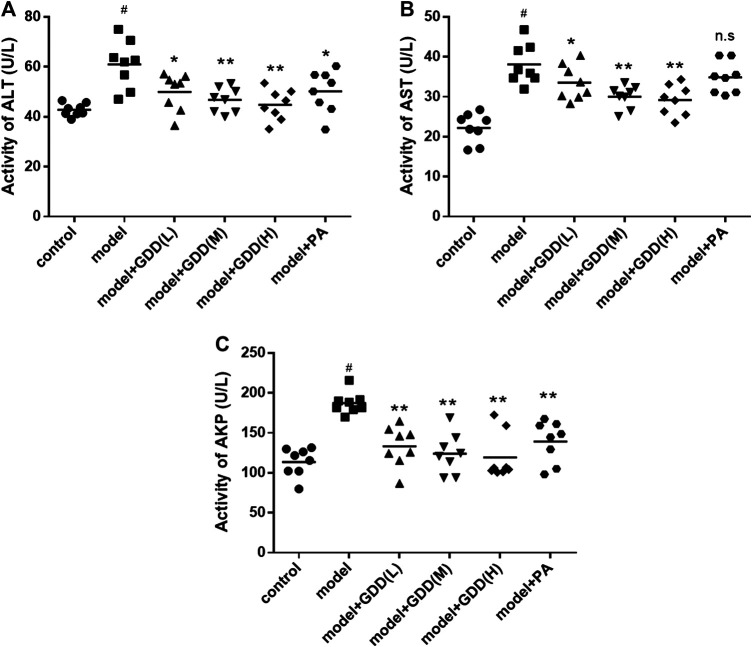
Effects of GDD on activity of ALT **(A)**, AST **(B)**, AKP **(C)** in the serum. ^#^ *p* < 0.01, compared with the control group; *p < 0.05, compared with model group; **p < 0.01, compared with model group; n.s, not significant.

To estimate the protective effect of GDD on the liver in rats induced by excessive copper, the indexes of AST, ALT, and AKP in serum were analyzed. The results in Figure 7 revealed a significant increase in the activities of serum ALT, AST, AKP in copper-laden model rats compared to normal controls (Figure 7, *p* < 0.01). The treatments with GDD at different doses and PA markedly lowered serum ALT activities (Figure 7A, *p* < 0.01 or *p* < 0.05). The treatment of model rats with GDD had significantly reduced serum AST activities (Figure 7B, *p* < 0.01 or *p* < 0.05), there was no significant difference in the AST activities of the PA treatment group and the model group. In addition, the treatment of rats with GDD and PA caused significant reductions in the AKP levels (Figure 7C, *p* < 0.01).

### Determination of Hepatic Indicators

**FIGURE 8:**
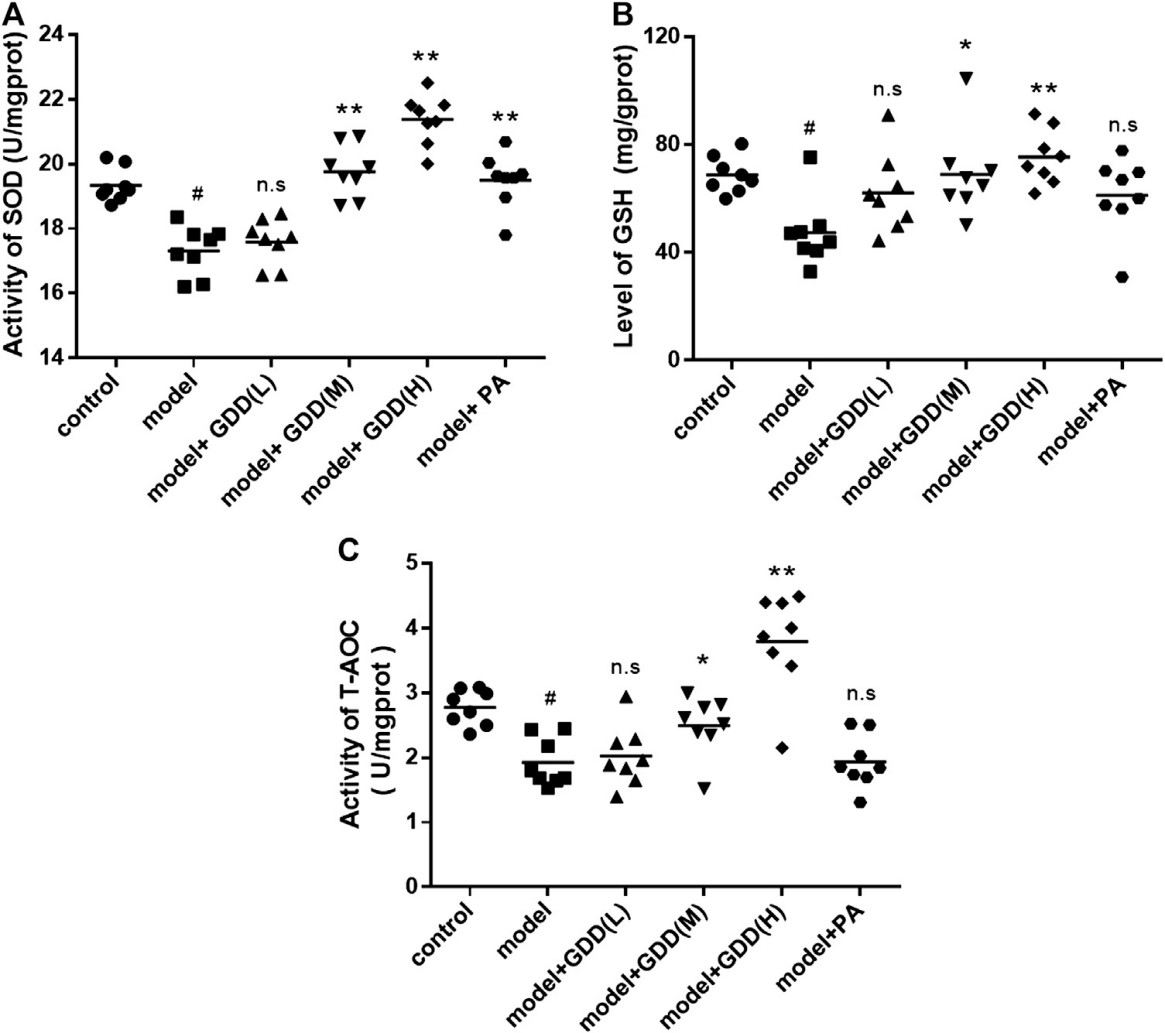
Effects of GDD on levels of SOD **(A)**, GSH **(B)**, T-AOC **(C)** in the livers. ^#^ *p* < 0.01, compared with the control group; *p < 0.05, compared with model group; **p < 0.01, compared with model group; n.s, not significant.

The results demonstrated that SOD activity in the liver of the model group was significantly lower than in the liver of the normal control group (Figure 8A, *p* < 0.01). The GDD-medium dose, GDD-high dose, and PA treatments increased SOD activity (*p* < 0.01). However, the activities of SOD in the liver were not significantly changed in the copper-laden rats treated with low-dose GDD (*p* > 0.05). GSH protects cells against peroxides, free radicals, and other toxic compounds. The levels of GSH in the tissues often decrease with increased local oxidative stress. The oxidative stress caused by excessive copper significantly reduced liver GSH levels. However, compared to the model rats, copper-laden rats administered GDD-medium dose and GDD-high dose treatments had significantly increased GSH levels (Figure 8B, *p* < 0.01 or *p* < 0.05). Interestingly, there was no significant difference in the GSH levels of the PA treatment group and the model group (*p* > 0.05). In addition, compared with the control group, the T-AOC level in the liver of the model group was markedly decreased (Figure 8C, *p* < 0.01). Compared to the model rats, the administration of medium-dose, and high-dose GDD treatments to copper-laden rats significantly increased T-AOC levels (*p* < 0.01 or *p* < 0.05). However, when treated with PA and a low dose of GDD, there was no significant difference in T-AOC levels compared with the model group (*p* > 0.05). These results indicating that GDD treatment significantly protected against hepatic indicators reduction.

### Histopathological Examination of the Liver

**FIGURE 9:**
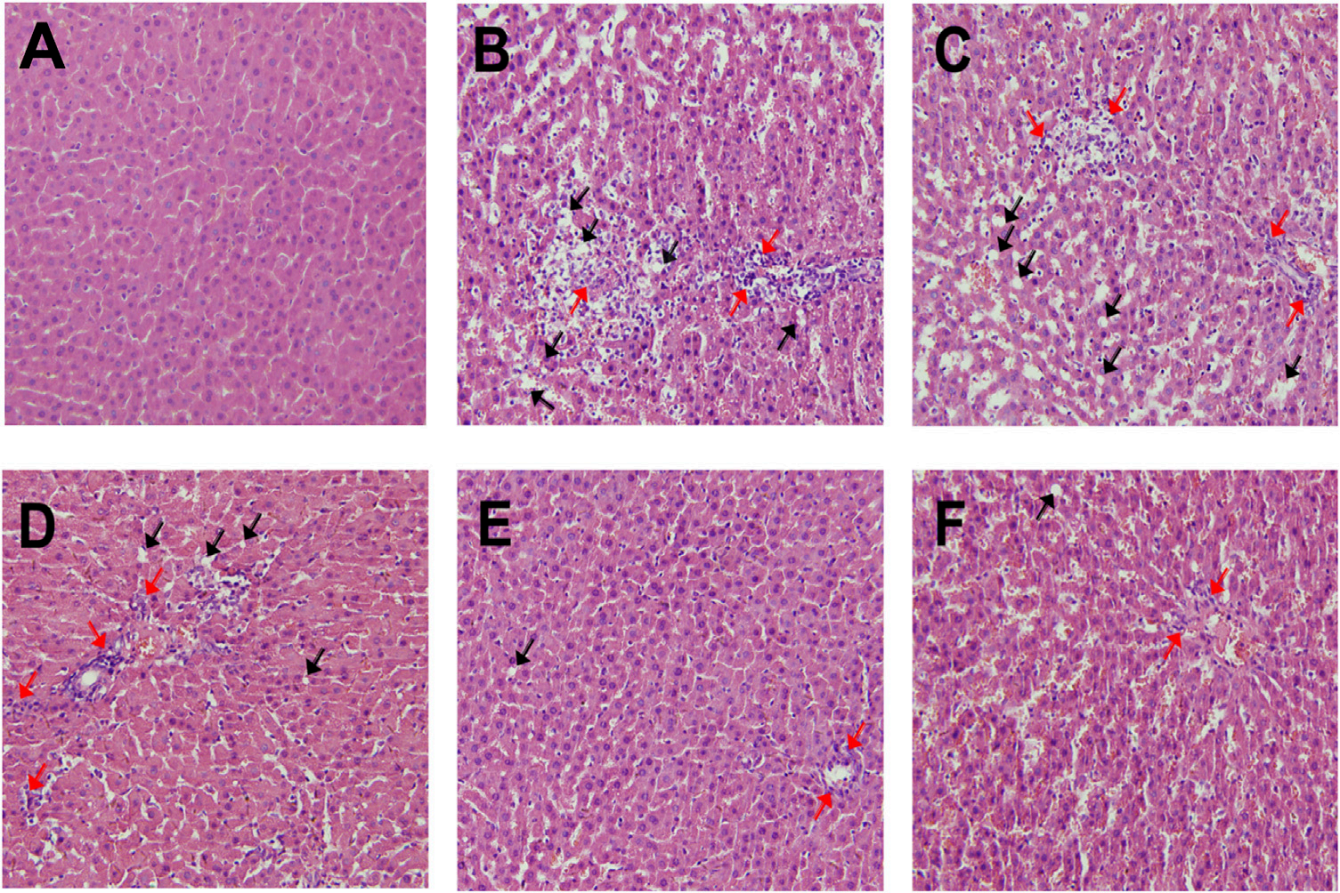
Effect of GDD on liver histology in rats. Representative photomicrographs with H&E staining (×200) reveal histopathological changes of liver. **(A)** control group; **(B)** Copper-laden model group; **(C)** low-dose GDD (0.65 g/kg/day); **(D)** medium-dose GDD (1.30 g/kg/day); **(E)** high-dose GDD (2.6 g/kg/day). **(F)** positive drug treatment (PA, 0.048 g/kg/day). Cytoplasmic vacuolations (black arrow), hepatocellular necrosis (red arrow).

On the basis of the histopathologic observations, the normal control group exhibited normal hepatocyte structure, orderly arrangement, clear contour, and no histological abnormalities (Figure 9A). In comparison, the copper-laden model group exhibited serious liver damage. The liver sections show that the hepatocytes exhibit clear cellular structure deterioration, disordered arrangement, cytoplasmic vacuolations, and necrocytosis as indicated by arrows in the figure (Figure 9B). The results showed that the severe liver injury in this model caused by the accumulation of copper ions modeled the clinical situation. The administration of varying doses of GDD significantly alleviated the abnormal structural disorder in the liver and cellular debris (Figure 9C-E). In addition, the rats administered a high dose of GDD showed better results than the positive control group (Figure 9F).

## Discussion

In HLD, the functional loss of copper-transporting P-type ATPase (ATP7B) impairs biliary copper excretion, leading to superabundant copper accumulation in the liver and oxidative stress (Gitlin, 2003; Kim et al., 2008). If HLD is diagnosed early, the prognosis is good with the current treatment for initial symptoms. As the disease progresses, early diagnosis of this disease is challenging, and treatment becomes more difficult, which makes the liver transplantation the only viable treatment option for patients (Boga et al., 2017). Currently, the dominant therapeutic strategy is to reduce the level of copper in patients and restore homeostasis. Nevertheless, these current approaches do not address the potential mutations in ATP7B that cause the disease. Hence, such treatments are expected to continue throughout the patient's life, with the focus on avoiding side effects. Existing therapies comprise treatment with either copper chelating agents or zinc salts. These drugs sufficiently control the symptoms. However, chelators could induce multiple severe toxicities, including significant bone marrow suppression, hypersensitivity reactions, degenerative changes in skin autoimmune disease, and nephrotoxicity (Ferenci et al., 2012). Zinc acetate is the first line of defense for treatment; it induces the expression of metallothionein in intestinal cells, reduces the absorption of copper by the intestines, and then subsequently captures the two metals, causing them to be excreted into the feces as mucosal cells fall off (Brewer and Yuzbasiyan-Gurkan, 1992). However, zinc acetate can not eliminate copper from overloaded tissues, although it is less toxic than chelating agents. Additionally, a study showed that more than 36% of Italian pediatric patients with HLD responded to the existing drugs, but the liver enzymes were not normalized. Clearly, new therapeutic strategies are required to treat HLD.

The amount of clinical practice and theoretical studies have indicated that GDD is a viable treatment option for HLD. However, the mechanism of GDD in the treatment of HLD is still unclear. A large number of studies have shown that TCM can promote the expulsion of heavy metals from the body through coordination of metal ions, and play a role in the expulsion of lead, copper, and calculus (Ma et al., 2010; Yang et al., 2010; Guo, 2019). The organic molecules such as flavonoids, anthraquinones, triterpenes, sugars, and amino acids in these natural drugs can mostly meet the conditions of forming complexes in structure, which can be used as ligands of complexes and metal ions to form complexes. The chemical components of detoxifying and spleen-reinforcing drugs mainly composed of amino acids can form stable complexes with Cd^2+^ and other toxic heavy metals, and the effect of eliminating toxic heavy metals in the body is better than EDTA-Zn salt (Zhu and Sun, 2000). The main Chinese herbal medicines for urolithiasis, such as *Lysimachia christinae Hance*, *Poria cocos* and *Polygonum cuspidatum*, contain flavonoids and alkaloids, can coordinate with Ca^2+^ to form complex, and promote calcium excretion and improve urinary calculi. Some studies have also shown that natural flavonoids genistein and kaempferol can complex with copper ions to increase their antioxidant activity *in vitro* (Ouyang, 2004). In addition, curcumin also has multipotent stone-fighting properties with few side effects and is a potential choice for the prevention and treatment of new or recurrent kidney stones (Li et al., 2019).

In order to further investigate the therapeutic effect of GDD on HLD, we used copper-laden hepatolenticular degeneration model rats as the research object, and conducted the study from two aspects: removing copper and liver protection. Our data indicated that when GDD was administered orally to the copper-laden rat model of HLD, the serum and hepatic copper levels were both lowered (Figures 4A,B). Moreover, this mechanism of copper removal may be related to the promotion of copper excretion in the urine and feces (Figures 4C,D). At the same time, in our study, we found that six active ingredients in GDD can also have a strong coordination reaction with Cu^2+^ (Figures 5, 6). The results of this study suggest that GDD may cooperate with Cu^2+^ and then excrete from urine and feces to play a role in copper elimination to treat HLD.

Copper is a trace element, which is mainly under either in the oxidized state (Cu^2+^) or reduced state (Cu^+^)in biological systems (Scheiber et al., 2014; Zhao et al., 2014). In addition, transitions between these states produce hydroxyl radicals, which are essential for the activity of different cellular enzymes involved in energy metabolism, respiration, and DNA synthesis (Tisato et al., 2010). However, redox reactions produce free radicals that can attack biological macromolecules, as a result, excess copper is highly toxic. Compounds isolated from natural products are considered an important source of new antioxidant drugs, with fewer side effects. In HLD patients, copper accumulation in the body with progressive liver damage caused by oxidative stress is the most main clinical symptom (Członkowska and Litwin, 2017). Modern pharmacological studies have shown that rhubarb extract can ameliorate alcohol-induced liver injury and down-regulate key markers of inflammation and oxidative stress in liver tissues (Neyrinck et al., 2016). Rutin, a flavonoid in *Lysimachia christinae* Hance, is an ingredient that could be helpful in the maintenance of the intracellular redox-homeostasis and may be effective against oxidative stress related secondary complications (Singh et al., 2019). Studies have also shown that curcumin, a representative component in turmeric has a certain protective effect on lipopolysaccharide/D-galactosamine-induced acute liver injury (Xie et al., 2017). Meanwhile, it is also reported that curcumin can prevent restraint stress-induced oxidative damage in the liver, brain, and kidney of rats (Samarghandian et al., 2017). Rhizoma Alismatis extract has also been verified to exhibit a protective effect against hepatocyte injury in Jian carp (Du et al., 2018). Berberine hydrochloride, a representative active ingredient in Coptis chinensis, can protect C2C12 cells from oxidative stress-induced damage by activating the Nrf2/HO-1 pathway (Choi, 2016). The total saponins of Panax notoginseng have also been shown to protect cardiomyocytes from endoplasmic reticulum stress and associated apoptosis by mediating intracellular calcium homeostasis (Chen et al., 2019). In light of these studies, we further investigated whether GDD protects against liver damage caused by excessive copper through inhibiting oxidative stress.

Elevated serum ALT, AST, and AKP activities are used generally to indicate the occurrence of hepatocellular injury (Kamei et al., 1986). Therefore, we next examined the above typical biomarkers for liver injury. The results showed that GDD dramatically reduced the elevated activities of serum ALT, AST, and AKP in copper-laden rats (Figure 7). Multiple reactive oxygen species (ROS) are caused by oxidative stress. Antioxidant enzymes (such as SOD) and non-enzymatic antioxidants (such as GSH) are deemed to be important factors of oxidative stress. SOD can reduce the production of free radicals and lipid peroxide and can even accelerate the clearance of these molecules, thus decreasing hepatocyte damage (Afonso et al., 2007; Ding et al., 2015). GSH serves as a reservoir of cysteine-counteracting ROS (De Matos and Furnus, 2000). SOD activities and GSH levels indirectly reflect the body’s ability to scavenge oxygen free radicals. Consequently, maintaining the appropriate levels of SOD, GSH, and T-AOC are regarded as essential to prevent and reduce oxidative damage. In the present study, the liver of copper-laden rats had decreased SOD activity as well as GSH and T-AOC levels, which is indicative of oxidative stress. However, medium-dose and high-dose GDD administration was significantly protected against the copper-induced reduction of SOD, GSH, and T-AOC (Figure 8). Interestingly, our data indicate that the treatment of copper-laden rats with PA cannot increase the activities of GSH and T-AOC in the liver. These results suggested that treatment with GDD could protect the liver from copper-induced hepatic oxidative stress. Meanwhile, the pathological changes in HE-stained liver tissue further confirmed the protective effect of GDD on liver injury in copper-laden rat models.

## Conclusion

The aim of the present study was to explore the therapeutic effect of GDD in HLD. Our study demonstrated that treatment with GDD effectively diminished the serum and hepatic copper levels through promoting urinary and fecal copper excretion in copper-laden rats. Additionally, for the first time, GDD was demonstrated to have protective effects against copper-induced hepatic injury in rats. This study highlights the exceptional value of GDD treatment for HLD. Therefore, GDD has a strong potential to become the safest and most effective medication to treat HLD patients.

## Data Availability

The raw data supporting the conclusions of this article will be made available by the authors, without undue reservation, to any qualified researcher.

## Glossary

AAS: atomic absorption spectrophotometry
AKP: alkaline phosphatase
ALT: alanine aminotransferase
AST: aspartate aminotransferase
BCA: total protein
GDD: Gandou Decoction
GSH: glutathione
H&E: hematoxylin-eosin
HLD: Hepatolenticular degeneration
HPLC: high-performance liquid chromatography
LOD: limit of detection
LOQ: limit of quantitation
PA: D-penicillamine
ROS: reactive oxygen species
RPA: relative peak area
RRT: relative retention time
SOD: superoxide dismutase
T- AOC: total antioxidant capacity
TCM: traditional Chinese medicine
UV-Vis: Ultraviolet-visible
WD: Wilson’s disease
