# Supplementary material for: Gandou Decoction Decreases Copper Levels and Alleviates Hepatic Injury in Copper-Laden Hepatolenticular Degeneration Model Rats
Source: Front Pharmacol. 2020 Oct 28;11:582390. doi: 10.3389/fphar.2020.582390 (PMC7970920; doi:10.3389/fphar.2020.582390)
Supplement: Supplementary file 1 [file Table1_v1.DOC]

Supplementary Material

**Supplementary Table 1 |** Detailed information of the crude drugs composed in GDD

| **Latin name** | **Chinese name** | **Place of Origin** | **[S](../../../../C:/Program%20Files%20(x86)/Youdao/Dict/8.9.3.0/resultui/html/index.html#/javascript:;)upplier** | **Bach number** | **Voucher Specimens Reserve** | **Voucher number** |
| --- | --- | --- | --- | --- | --- | --- |
| **Rheum palmatum L.** | Da huang | [Gansu](../../../../C:/Program%20Files%20(x86)/Youdao/Dict/8.9.3.0/resultui/html/index.html#/javascript:;), China | Beijing Tongrentang Pharmacy Co., Ltd. | 20180106 | Anhui University of Chinese Medicine, Hefei, China | 18056 |
| **Coptis chinensis Franch.** | Huang lian | [Sichuan](../../../../C:/Program%20Files%20(x86)/Youdao/Dict/8.9.3.0/resultui/html/index.html#/javascript:;), China | Beijing Tongrentang Pharmacy Co., Ltd. | 20180101 | Anhui University of Chinese Medicine, Hefei, China | 18031 |
| **Curcuma longa L.** | Jiang huang | Sichuan, China | Beijing Tongrentang Pharmacy Co., Ltd. | 220180120 | Anhui University of Chinese Medicine, Hefei, China | 18059 |
| **Lysimachia christinae Hance** | Jin qian cao | Sichuan, China | Beijing Tongrentang Pharmacy Co., Ltd. | 20180113 | Anhui University of Chinese Medicine, Hefei, China | 18070 |
| **Alisma orientale (Sam.) Juzep.** | Ze xie | Sichuan, China | Beijing Tongrentang Pharmacy Co., Ltd. | 20180107 | Anhui University of Chinese Medicine, Hefei, China | 18052 |
| **Panax notoginseng (Burk.) F. H. Chen** | San qi | Yunnan, China | Beijing Tongrentang Pharmacy Co., Ltd. | 20180109 | Anhui University of Chinese Medicine, Hefei, China | 18067 |
